# Genomic characterisation, chromosomal assignment and *in vivo* localisation of the canine High Mobility Group A1 (HMGA1) gene

**DOI:** 10.1186/1471-2156-9-49

**Published:** 2008-07-23

**Authors:** Claudia Beuing, Jan T Soller, Michaela Muth, Sigfried Wagner, Gaudenz Dolf, Claude Schelling, Andreas Richter, Saskia Willenbrock, Nicola Reimann-Berg, Susanne Winkler, Ingo Nolte, Jorn Bullerdiek, Hugo Murua Escobar

**Affiliations:** 1 Clinic for Small Animals and Research Cluster of Excellence "REBIRTH", University of Veterinary Medicine Hanover, Bischofsholer Damm 15, 30173 Hanover, Germany; 2 Centre for Human Genetics, University of Bremen, Leobener Str ZHG, 28359 Bremen, Germany; 3 Institute of Animal Genetics, Nutrition and Housing, University of Berne, Berne, Switzerland; 4 Department of Animal Sciences, Swiss Federal Institute of Technology Zurich and Vetsuisse Faculty Zurich, University of Zurich, Zurich, Switzerland

## Abstract

**Background:**

The high mobility group A1 proteins (HMGA1a/HMGA1b) are highly conserved between mammalian species and widely described as participating in various cellular processes. By inducing DNA conformation changes the HMGA1 proteins indirectly influence the binding of various transcription factors and therefore effect the transcription regulation. In humans chromosomal aberrations affecting the *HMGA1 *gene locus on HSA 6p21 were described to be the cause for various benign mesenchymal tumours while high titres of HMGA1 proteins were shown to be associated with the neoplastic potential of various types of cancer. Interestingly, the absence of HMGA1 proteins was shown to cause insulin resistance and diabetes in humans and mice.

Due to the various similarities in biology and presentation of human and canine cancers the dog has joined the common rodent animal model for therapeutic and preclinical studies. Accordingly, the canine genome was sequenced completely twice but unfortunately this could not solve the structure of canine *HMGA1 *gene.

**Results:**

Herein we report the characterisation of the genomic structure of the canine *HMGA1 *gene consisting of 7 exons and 6 introns spanning in total 9524 bp, the *in vivo *localisation of the HMGA1 protein to the nucleus, and a chromosomal assignment of the gene by FISH to CFA12q11. Additionally, we evaluated a described canine *HMGA1 *exon 6 SNP in 55 Dachshunds.

**Conclusion:**

The performed characterisations will make comparative analyses of aberrations affecting the human and canine gene and proteins possible, thereby providing a basis for revealing mechanisms involved in HMGA1 related pathogenesis in both species.

## Background

The high mobility group A (HMGA) proteins are small chromatin associated non-histone proteins named according to their characteristic motility in acid-urea polyacrylamide gel electrophoresis. The protein family consists of the three proteins HMGA1a, HMGA1b and HMGA2 which are encoded for by two different genes (HMGA1 and HMGA2). The functional motifs of these proteins, named AT-hooks, bind to the minor groove of DNA causing conformational changes of the DNA molecule. On genomic level these structural changes influence the binding of various transcription factors and thus indirectly influence the transcription regulation, which classifies the HMGA proteins as so called architectural transcription factors (for detail see [1]).

In previous studies we characterised the canine HMGA1 cDNAs and proteins and in comparative analyses of these molecules showed that they are highly conserved between different mammalian species. The observed number of amino acid changes seen across mammalian species (cattle, dog, hamster, horse, mouse, pig, and rat) vary between 0 to 3 when compared to the human molecules [2-10]. Interestingly, only the canine HMGA1 proteins are 100% identical to their respective human counterparts [11].

The HMGA1 proteins are well known to play a significant role in the pathogenesis of various diseases including cancer. In humans, chromosomal aberrations affecting the HMGA1 gene locus on HSA 6p21 were described for various benign mesenchymal tumours, e.g. endometrial polyps, lipomas, pulmonary chondroid hamartomas, and uterine leiomyomas [12-14]. The observed aberrations are supposed to lead to an up-regulation of the *HMGA1 *gene in the affected tumours, as opposed to adult healthy tissues where *HMGA *gene expression is low or hardly measurable [9,15,16]. In malignant neoplasias *HMGA1 *expression is reported to be associated with an aggressive behaviour of tumours. Accordingly, *HMGA1 *overexpression was detected in various malignancies including thyroid, lung, prostatic, pancreatic, uterine cervical, and colorectal carcinoma [17-22]. Thus *HMGA *expression is supposed to present a powerful diagnostic and prognostic molecular marker due to the described correlation between *HMGA *expression and tumour aggressiveness.

Whilst overexpression of *HMGA1 *is clearly associated with cancerogenesis the disruption of the *HMGA1 *gene and thus induced loss of *HMGA1 *expression shows significant pathogenic effects. Heterozygous and homozygous Hmga1 knock-out mice develop cardiac hypertrophy combined with hematologic malignancies e.g. B cell lymphoma and myeloid granuloerythroblastic leukemia [23]. Additional research with Hmga1 knock-out mice targeting diabetes presented by Foti et al. (2005) showed that loss of Hmga1 expression is clearly associated with significantly decreased insulin receptor expression and thus causes a characteristic diabetes type 2 phenotype in mice [24].

The various similarities in presentation and biology of numerous canine and human diseases including cancer suggest similar mechanisms to be involved in the respective pathogenic events. Accordingly, at least a dozen distinct canine cancers are hypothesized to be appropriate models for their human counterparts, among those osteosarcoma, breast carcinoma, oral melanomas, lung carcinomas and malignant non-Hodgkin's lymphomas [25].

The characterization of disease related genes and their protein biology will allow for comparative studies to reveal the molecular mechanisms involved therein and serve as a basis for future clinical studies.

## Results and discussion

The *HMGA1 *gene and its proteins HMGA1a and HMGA1b are described as regulating multiple cellular processes and are widely reported to be associated with various diseases including diabetes and cancer. In previous studies we characterised the canine *HMGA1 *cDNAs and proteins completely and did comparative analyses of these molecules to the respective counterparts of different species and showed high evolutionary conservation. The fact that several canine and human cancer types show striking similarities in presentation and biological behaviour, e.g. spontaneous occurrence and metastasis patterns, strongly suggests similar mechanisms to be involved in the respective pathogenic events of both species. Thus, various canine tumours are currently used as models for several human cancer types. Accordingly, comprehension of the canine gene and its gene products is precondition for comparative analyses, allowing the revelation of molecular effects involved in these pathogenic presentations. Understanding and comparison of the respective genes will thus benefit both species. The exact mechanism for the emergence of the pathogenic effects caused by chromosomal aberrations affecting the human *HMGA1 *gene in benign mesenchymal tumours, e.g. endometrial polyps, lipomas, pulmonary chondroid hamartomas, and uterine leiomyomas [12-14] are not completely understood. However, it is currently supposed that the aberration causes up-regulation of the *HMGA1 *gene in the affected neoplasias. The principal aim of the study was to characterize the genomic structure of the canine *HMGA1 *gene allowing the comparison of its genomic structure to the counterparts of other mammals and thus allowing a further evaluation of evolutionary conservation of the gene and a comparative analysis of chromosomal aberrations in both species. Additional aims were the *in vivo *localization of the canine HMGA1 protein and the evaluation of a previously described point mutation which causes a disrupted protein.

### Genomic structure, BAC Screening and FISH

**Figure 1:**
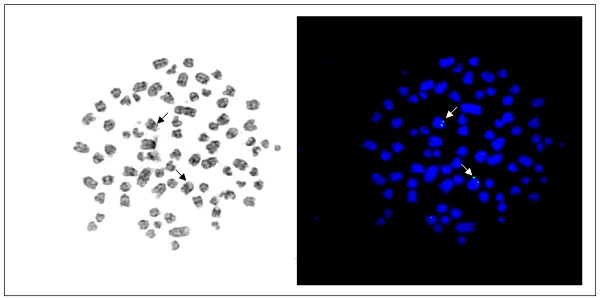
**FISH-Mapping of the canine *HMGA1***. Canine metaphase spread after GTG-banding (left) and the same metaphase after fluorescence *in situ *hybridisation with BAC MGA 572 P20 K12 RC showing signals on both chromosomes 12 (right).

A canine *HMGA1 *genomic PCR reaction was established and used for screening of a canine BAC for identification of the canine HMGA1 gene locus by FISH. The verified BAC 572 P20 K12 RC was used for FISH experiments. Ten well spread metaphases were analysed and showed signals on both chromatides of both chromosomes CFA 12q11 (Figure 1). The chromosomal localisation was done following the nomenclature established by Reimann et al. [26]. Existing painting probe based synteny studies and RH analyses [27] indicated that the canine CFA 12 shares homology with the human chromosome 6 on which the *HMGA1 *gene is located at HSA 6p21. Chromosomal aberrations affecting CFA 12 are not or barely reported to be significantly associated with canine neoplasias [28,29]. While previous studies reported the localization of a HMGA1 gene positive BAC to CFA 23 [30], the performed *in silico *analyses and the recently published canine genome assembly [31] support the herein described assignment of the canine *HMGA1 *gene to CFA 12q11 by FISH described in this study. Comparative chromosomal *in silico *analyses using the "Evolutionary Highway"  showed similar results.

**Figure 2:**
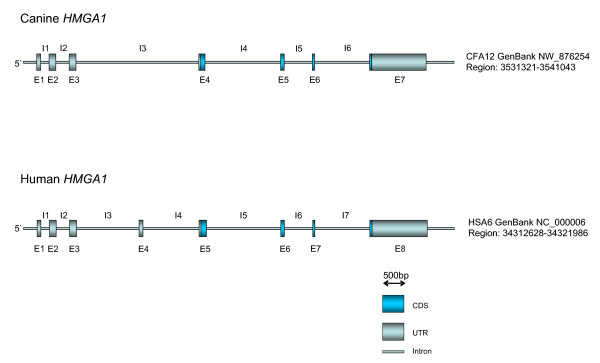
**Genomic structure of the canine *HMGA1 *gene**. Detailed structure of the genomic organisation of the canine *HMGA1 *gene.

The genomic structure of the canine *HMGA1 *gene consists in total of the 7 exons and 6 introns. Overall the canine *HMGA1 *gene spans 9524 bp. The exon/intron structure, size and the homologies to their human counterparts were analysed and defined (Figure 2, Table 1). The total identity to the corresponding human region is 62.8%. In detail, the identities of the exons vary between 74.6% and 97.8% to their human counterpart, while the introns show identities between 58.9% and 92.4% (for details see Table 1). The newly characterized sequences combined with the analyses performed *in silico *revealed that the exon 4, which exists in humans, is missing on genomic level in the canine genome. This exon 4 deletion also exists in the mouse genome and affects the respective mRNAs of both species in their 5' UTR. As the genomic characterization of the canine HMGA1 gene was not available when the exons were named previously, the numbering at that time was based on the respective human exon numbers as defined by Friedmann et al. [32]. Consequently, as it is now known that the canine genomic sequence is lacking an equivalent to human exon 4, the previously used canine exon numbering should be revised with the then named canine exon 5 now being canine exon 4 and so on (Figure 2, Table 1). However, a part of intron 2 remains unsequenced due to an extensive CG repeat which also exists in the human counterpart (90%CG), and only the number of nucleotides (311 bp) could be identified. The genomic sequences were submitted to the NCBI database (bankit1078465, bankit1078536, bankit1078968).

**Table 1:** Detailed analysis of the canine *HMGA1 *gene genomic elements

**Element of canine *HMGA1 *gene**	**Size in bp**	**Identity to human counterpart in % (GenBank **NC_000006))
**Total gene**	9524	62.8
		
Detail exons/introns (revised numbering)*		
Exon 1	94	97.8
Intron 1	196	92.4
Exon 2	164	95.8
Intron 2	311	-
Exon 3	162	74.6
Intron 3	3096	58.9
Exon 4 (5)	179	93.9
Intron 4 (5)	1761	51.1
Exon 5 (6)	84	96.4
Intron 5 (6)	584	57.5
Exon 6 (7)	51	94.1
Intron 6 (7)	1459	58.1

Exon 7 (8)	1386	75.4

Identity comparison of the genomic elements of the canine *HMGA1 *gene with its respective human counterparts.

### Exon 6 SNP evaluation

**Figure 3:**
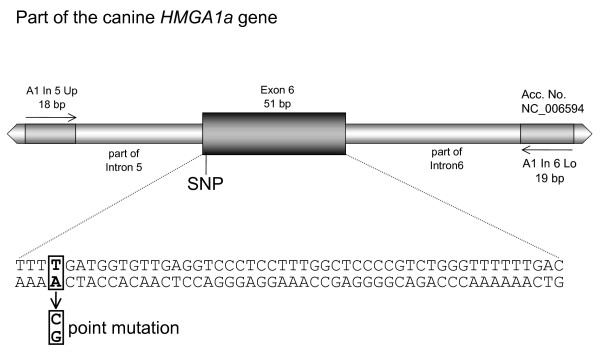
**Position of the evaluated Dachshund point mutation**. Strategic position of the evaluated point mutation screened in 55 Dachshunds.

While characterising the canine *HMGA1 *gene we screened twelve different canine breeds for point mutations affecting the protein coding region. A Dachshund sample showed a transition from A to G in exon 6 (according to revised exon numeration) leading to an amino acid exchange from threonine to alanine causing a mutated HMGA1 protein [9]. To elucidate if the observed exchange is frequently existent in the Dachshund population we screened 55 Dachshunds for the respective mutation (Figure 3). The results obtained by sequencing and restriction fragment analysis clearly showed that the previously found mutation is a rare event, as none of the screened 55 Dachshunds showed the mutation. Thus our findings suggest that the previously found aberrant *HMGA1 *allele leading to a mutated protein form is unlikely to play a major role in HMGA1 pathogenesis in Dachshunds.

In general, different species show significant differences considering the number and probability of described SNPs. This fact surely is directly dependent on total numbers of studies and sequencing reactions performed for the different species. While in 2001 Sachidanandam et al. [33] detected 1.42 million SNPs in the human genome with one SNP per 1.9 kb the currently estimated total number reported SNPs in the public databases is approx. 9 million for the human genome [34]. For the dog Lindblad-Toh et al. reported 2.5 million SNPs, whereas the probability differs depending on the breed between one SNP per 1500 bp and 900 bp [31]. Comparable to the human genome the total numbers of reported SNPs in the other different species is expected to increase significantly according to the performed research efforts, leading to increased knowledge of effects caused by SNPs in general.

### HMGA1 *in vivo* localization

**Figure 4:**
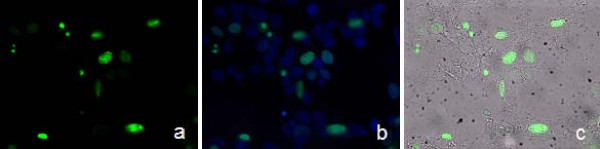
***In vivo *localisation of the canine HMGA1 protein**. *In vivo *localization of a canine HMGA1a-GFP fusion protein in culture canine MTH53A cells, 24 h posttranslational. a) GFP expression in canine mammary cell line MTH53A, b) DAPI fluorescent staining of cell nuclei, merged GFP and DAPI image, c) merged GFP and transmitted light image (magnification ×400).

The *in vivo *localization of the canine HMGA1 proteins via expression of a canine HMGA1a-GFP fusion protein showed that equivalently to its human counterpart the protein is located in the nucleus (Figure 4). Proteins of the HMGA family are described to be architectural transcription factors, and thus a localisation in the nucleus seems obvious. However, further localisation and function of these proteins seem to be very likely, due to the fact that application of recombinant HMGA1 proteins to porcine cartilage cells *in vitro *showed significant increase of cell proliferation (Richter et al. accepted for publication). For a further member of the HMG proteins called HMGB1 the existence of an extracelluar function was recognised only a long time after its initial characterisation as an architectural transcription factor, revealing a direct influence of extracellular HMGB1 on metastatic events [35-37]. Thus, we suppose that a similar mechanism could also exist for HMGA proteins and are currently working towards its identification.

## Conclusion

Knowledge about the structure of genes and proteins is precondition to use them as potential therapeutic targets, markers or for revealing mechanisms involved in relevant pathogenic events. The canine and human HMGA genes and proteins have widely been shown to be involved in various diseases especially in cancer. Due to the numerous reasons for using the dog as a model system for human cancer research the characterisation of canine genes and proteins is of special interest. The performed characterisations of the canine HMGA1 gene and proteins will allow performing comparative analyses of aberrations affecting the human and canine genes and proteins as basis for revealing mechanisms involved in HMGA1 related pathogenesis in both species.

## Methods

### BAC library screening

A PCR reaction for the use in PCR-based screening of the *Canis familiaris *DogBAC library (Schelling et al., 2002) (Institute of Animal Genetics, Nutrition and Housing, University of Berne, Berne, Switzerland) for a BAC clone containing *HMGA1 *was established using canine genomic DNA derived from blood. The primers A1In5up (5' GGCATCCGGTGAGCAGTG 3') and A1In6lo (5' CAGGCAGAGCACGCAGGAC 3') were designed using GeneBank sequences AY366395 &NW_876254. PCR parameters were: 95°C for 5 min, followed by 30 cycles of 95°C 30 sec, 59.3°C 30 sec, 72°C 30 sec, and a final elongation of 72°C for 10 min. The corresponding 201 bp PCR product was cloned into the pGEM-T Easy vector system (Promega, Mannheim, Germany) and verified by sequencing. The DNA contigs and alignments were done with Lasergene software (DNAstar, Madison, USA) and various sequences from the NCBI database (AY366395, NW_876254). The verified BAC clone MGA 572 P20 K12 RC was used as probe for the following FISH experiments.

### Slide Preparation

1 ml of canine whole blood was incubated for 72 h in Chromosome Medium B (Biochrom, Berlin, Germany). Subsequently, colcemide (0.1 μg/ml) (Biochrom, Berlin, Germany) was added for 2 hours. The cells were centrifuged at 135 × g for 10 min and incubated for 20 min in 0.05 M KCl. Finally the cells were fixed with methanol/glacial acetic acid. This suspension was dropped on ice-cold slides and dried for at least 7 days at 37°C. The chromosomes were stained by GTG banding for karyotype description. Prior to use in FISH investigations, the slides were destained with 70% ethanol.

### Fluorescence *in situ* Hybridization

MGA 572 P20 K12 RC BAC-DNA was digoxigenin labelled (Dig-Nick-Translation-Kit, Roche, Mannheim, Germany). The hybridization mixture contained 200 ng probe, 40 ng ssDNA, 600 ng sonicated dog DNA, 2 × SSC, 2 × SSPE, 50% formamide and 10% dextran sulfate. 50 μl of this mixture were applied to each slide and the cover slips were sealed with rubber cement. Probe and chromosomes were denatured at 75°C on an Eppendorf Thermocycler gradient, using the *in situ *adapter. Afterwards, the slides were incubated in a moist chamber at 37°C over night. Cover slips were carefully removed and the slides were incubated in 0.1 × SSC at 61°C and 1 × PBS at RT. Slides were then covered with 100 μl non fat dry milk (NFDM) for 20 min. at 37°C in a moist chamber. For signal detection 100 μl NFDM containing 3 μg of Anti-Digoxigenin-Rhodamine, Fab fragments (Roche, Mannheim, Germany), were added to each slide and again incubated for 20 min at 37°C in a moist chamber, followed by washes with 1 × PBS, 3 × 3 min. at RT. Slides were air dried before chromosomes staining was performed with 25 μl of Vectashield Mounting Medium with DAPI (Vector Laboratories, Burlingame, CA, USA)

Ten well spread metaphases were examined indicating a signal on CFA 12q11 on both chromatids of both chromosomes CFA 12q11 (Fig. 1). The determination of chromosomes follows the nomenclature of the canine karyotype as described previously [26].

### Genomic characterisation

For genomic characterisation of the canine *HMGA1 *gene the missing parts were amplified by PCR on the screened BAC clone MGA 572 P20 K12 RC. For the missing part 1 a 858 bp fragment (bankit 1078968) was generated with primer pair A1_6640-6997_upa (5'-GGCGCGGCTCCAAGAA-3'), A1_6_lo_2 (5'-CCAACAGAGCCCTGCAAA-3'), a 1879 bp fragment (bankit 1078465 for the missing part 2 was generated by the primer pair A1_8864-10549_upa (5'-GTCTCACCGTCTGGAGAAT-3'), A1_8864-10549_loa (5'-TCACCGGAGGCTGCTT-3') and for the third missing part a 979 bp fragment (bankit 1078536) was generated with primer pair A1_11223-11834_upa (5'-CTGAGCCCATGCCAGATAA-3'), A1_11223-11834_loa (5'-AGAGATCCCTGCCGTAGT-3'). The obtained PCR products were separated on a 1.5% agarose gel, recovered with QIAquick Gel Extraction Kit (QIAGEN, Hilden, Germany), cloned in pGEM-T Easy vector system (Promega, Mannheim, Germany) and sequenced for verification. The final genomic canine *HMGA1 *contig and the identity alignments were created with Lasergene software (DNAStar, Madison, USA) with the generated sequences from the cloned cDNAs described previously and various sequences from the NCBI database derived from the canine genome sequencing (AY366394, AY366395, AY366396, NM_001003387, NW_876254).

### SNP screening

Genomic DNA was isolated from the collected 55 Dachshunds samples using the QiaAmp kit (QIAGEN, Hilden, Germany). A specific genomic PCR using the primer pair A1In5up (5' GGCATCCGGTGAGCAGTG 3') and A1In6lo (5' CAGGCAGAGCACGCAGGAC 3') was established allowing the amplification of the complete exon 6 and flanking regions of intron 5 and 6, respectively (Figure 3). In detail the PCRs were performed in a 25 μl volume containing 0.5 μM of both primers (MWG Biotech, Martinsried, Germany), 0.1 mM of each dNTP (Invitrogen, Karlsruhe, Germany) 0.6 units Taq-DNA polymerase (Promega, Mannheim, Germany), 1.5 mM MgCl_2 _(Promega, Mannheim, Germany), PCR buffer (Promega, Mannheim, Germany) and 2.5 μl template DNA, containing averaged 26.5 ng/μl.

After an initial denaturation step of 5 min at 95°C, the amplification followed in 30 cycles (30 sec. at 95°C, 30 sec at 59.3°C and 30 sec at 72°C). To complete, a final elongation step for 10 min. at 72°C completed the process. The obtained PCR products were purified using the QIAquick PCR Purification Kit (Qiagen, Hilden, Germany), directly sequenced by MWG Biotech (Martinsried, Germany), and additionally digested enzymatically with AluI (Fermentas, St. Leon-Rot, Germany). The occurrence of the described SNP creates a new restriction site for the enzyme AluI (5' AG^▼^CT 3'). Thus, a digestion with AluI cuts the 201 bp PCR product in two fragments of 69 bp and 132 bp, respectively allowing a verification of the sequencing results.

### HMGA1 *in vivo *localisation

For the HMGA1 *in vivo *localisation the protein coding sequence of the canine HMGA1a was amplified by PCR using primer pair EcoR1_IY-upATG (5'-CGGAATTCCACCATGAGCGAGTCGAGCTCGA-3'), BamH1_IY-loSTOP (5'-CGGGATCCTCACTGCTCCTCTTCGGAGGACT-3'). The obtained PCR products were separated on a 1.5% agarose gel, recovered with QIAquick Gel Extraction Kit (QIAGEN, Hilden, Germany), ligated into the pEGFP-C1 vector plasmid (BD Bioscience Clontech) and sequenced for verification.

Cells from canine mammary tumour cell line MTH53a were cultivated using medium 199 (Invitrogen, Karlsruhe, Germany) supplemented with 20% FCS, penicillin, and streptomycin. The transfection was performed according to the manufacturer's instructions using 3 μl FugeneHD reagent (Roche, Mannheim, Germany) in 100 μl PBS (without Mg^2+^) containing 2 μg of recombinant pEGFP-C1-HMGA1a. After treatment, the cells were incubated for 48 hours in the culture media. The uptake and expression of DNA was verified by fluorescence microscopy.

## Authors' contributions

CB: collected the Dachshund samples and performed the point mutation screening, JB: head of the centre for human genetics, took part in the conception design of the study, GD: constructed the screened BAC library, JTS: *in silico *analyses and construction of the *HMGA1 *gene structure, MM: construction of expression vectors for the *in vivo *localisation, HME: principal study design, IN: head of the small animal clinic, took part in the conception design of the study, NR-B: karyotyping, AR: transfection of cells for in vivo localisation, CS: screening of the canine BAC library, SiW: molecular cloning of the newly characterised HMGA1 fragments, SaW: supervision point mutation screening, SuW: performed the FISH experiments.
